# Congenital adrenal hyperplasia, disorders of sex development, and infertility in patients with *POR* gene pathogenic variants: a systematic review of the literature

**DOI:** 10.1007/s40618-022-01849-9

**Published:** 2022-07-17

**Authors:** C. Gusmano, R. Cannarella, A. Crafa, F. Barbagallo, S. La Vignera, R. A. Condorelli, A. E. Calogero

**Affiliations:** Endocrinology, Department of Clinical and Experimental Medicine, University of Catania, Via S. Sofia 78, 95123 Catania, Italy

**Keywords:** Congenital adrenal hyperplasia, CAH, Testicular adrenal rest tumor, TART, POR, Sperm parameters, Fertility, Disorders of sex development, DSD

## Abstract

**Background:**

P450 oxidoreductase (POR) deficiency (PORD) is characterized by congenital adrenal hyperplasia (CAH) and disorders of sex development (DSD) in both sexes. PORD can also associate with skeletal defects. However, the prevalence of these phenotypes is unknown.

**Aim:**

To evaluate the prevalence of CAH, DSD, and infertility of patients with *POR* gene pathogenic variants by a systematic review of the literature.

**Methods:**

The literature search was performed through PubMed, MEDLINE, Cochrane, Academic One Files, Google Scholar, and Scopus databases. All studies reporting information on CAH, DSD, testicular adrenal rest tumor (TARTs), and fertility in patients with *POR* gene pathogenic variants were included. Finally, the prevalence of abnormal phenotypes was calculated.

**Results:**

Of the 246 articles initially retrieved, only 48 were included for a total of 119 (46 males and 73 females) patients with PORD. We also included the case of a male patient who consulted us for CAH and TARTs but without DSD. This patient, found to be a carrier of combined heterozygous *POR* mutation, reached fatherhood spontaneously. All the patients found had CAH. The presence of DSD was found in 65.2%, 82.1%, and 82.1% of patients with compound heterozygosity, homozygosity, or monoallelic heterozygous variants, respectively. The prevalence was significantly higher in females than in males. The prevalence of TARTs in patients with PORD is 2.7%. Only 5 women with PORD became pregnant after assisted reproductive techniques and delivered a healthy baby. Except for the recently reported proband, no other studies focused on male infertility in patients with *POR* gene variants.

**Conclusion:**

This systematic review of the literature reports the prevalence of CAH, DSD, and TARTs in patients with PORD. The unknown prevalence of *POR* gene pathogenetic variants and the paucity of studies investigating fertility do not allow us to establish whether PORD is associated with infertility. Further studies on both women and men are needed to clarify this relationship.

## Introduction

Congenital adrenal hyperplasia (CAH) describes a group of autosomal recessive disorders of cortisol biosynthesis with varying levels of severity [1]. Indeed, the clinical phenotype is typically classified as a classic form (the most severe one), and non-classic that is mild or late onset. Disorders of sex development (DSD) depend on the affected gene that cause CAH but also on sex proband. The presence of salt-wasting, postnatal virilization, sex steroid deficiency, hypertension, and other features, such as skeletal defects, are caused by the specific gene that is mutated [1]. Among the long-term complications of CAH, infertility is frequent in both female and male patients [2–8]. Male patients may experience the growth of testicular adrenal rest tumors (TARTs), which can cause intra-testicular compression of efferent seminiferous tubules in turn affecting spermatogenesis [9].

CAHs have a specific hormonal pattern based on the enzymatic dysfunction that causes it. The *CYP21A2* genotype is the main, but not the only, determinant of the phenotype in patients with 21α-hydroxylase deficiency (21α-OHD) [10]. In some patients suspected of having a 21α-OHD, no pathogenic variants were found in one or both alleles even after complete sequencing of the *CYP21A2* gene [11]. Other patients have pathogenetic variants of the CYP21A2 gene, but the genotype does not fully match the phenotype. These apparent discrepancies between genotype and phenotype suggest the presence of other genetic factors including modifier genes that can modulate the clinical expression of some aspects of 21α-OHD [10].

In humans, P450 oxidoreductase (POR) deficiency (PORD) causes an unusual and rare form of CAH, whose exact incidence is not known [12]. POR is an 82-kDa membrane-bound protein containing 680 residues encoded by a 32-kb gene containing 15 exons mapping on chromosome 7q11.2 [13]. It is necessary for the metabolic activity of P450 cytochrome enzymes including CYP17A1, CYP21A2, CYP19A1, and CYP51A1 [14]. Consequently, PORD can affect the function of these enzymes with different phenotype based on the residual enzymatic activity. As an example, studies on the CYP17A1, the steroidogenic enzyme that catalyzes both 17α-hydroxylase and 17,20 lyase activities [15], show that the levels of some P450 activities are determined, at least in part, by the stereochemistry of the interaction of POR with the cytochrome P450 [13]. In the case of CYP17A1, the 17,20 lyase reaction, but not the 17α-hydroxylase reaction, is very sensitive to this stereochemistry, as shown by three lines of evidence [13]. First, variants of basic residues in the redox-partner binding site of CYP17A1 selectively reduce the 17,20 lyase activity [16]. Second, cytochrome b5 acts as an allosteric factor to promote the interaction of P450c17 with POR, selectively increasing 17,20 lyase activity [17–19]. Third, phosphorylation of CYP17A1 selectively increases the 17,20 lyase activity of CYP17A1 [19–21].

PORD phenotype is characterized by DSD in both sexes and is often associated with skeletal defects [13, 22]. The latter, known as Antley–Bixler syndrome (ABS), is characterized by craniosynostosis, brachycephaly, radio-ulnar or radio-humeral synostosis, bowed femora, arachnodactyly, midface hypoplasia, proptosis, and choanal stenosis. ABS is transmitted with an autosomal recessive mechanism by *POR* pathogenic variants and with an autosomal dominant mechanism by gain-of-function variants of the *fibroblast growth factor receptor 2* (*FGFR2*) gene [23]. No definitive data are available on TARTs and fertility in male patients with recessive PORD.

Therefore, this study aimed to systematically review the literature to gather all the available information on gender and genotype-related prevalence of CAH, DSD, TARTs, and fertility in patients with heterozygous or homozygous *POR* gene variants. In the resulting database, we added also the case of a male patient with CAH and TARTs but without DSD. He resulted in being a carrier of a combined heterozygous *POR* pathogenic variant and achieved fatherhood spontaneously.

## Systematic review of the literature

### Methods

#### Sources

Data for the systematic review were independently extracted by C.G. and R.C. A systematic search was performed through PubMed, MEDLINE, Cochrane, Academic One Files, Google Scholar, and Scopus databases, from the beginning of each database through May 22, 2021. The search strategy was based on the combination of the following Medical Subjects Headings (MeSH) terms and keywords, using “AND” between each MeSH search term: “P450 oxidoreductase” AND “congenital adrenal hyperplasia”, “P450 oxidoreductase” AND “DSD”, “P450 oxidoreductase” AND “homozygosity”, “P450 oxidoreductase” AND “heterozygosity”, “P450 oxidoreductase” AND “pregnancy”. Additional manual searches were made using the reference lists of relevant studies. Only articles available in English full-text have been included.

#### Study selection

All studies that reported the clinical phenotype of patients with *POR* gene variants were included. In particular, we focused on the presence of CAH, DSD (defined as virilization in female patients, under-masculinization in male patients, and abnormalities of the reproductive system in both sexes), TARTs, and fertility. Review articles and studies on experimental animals were excluded.

#### Description of the proband

**Fig. 1:**
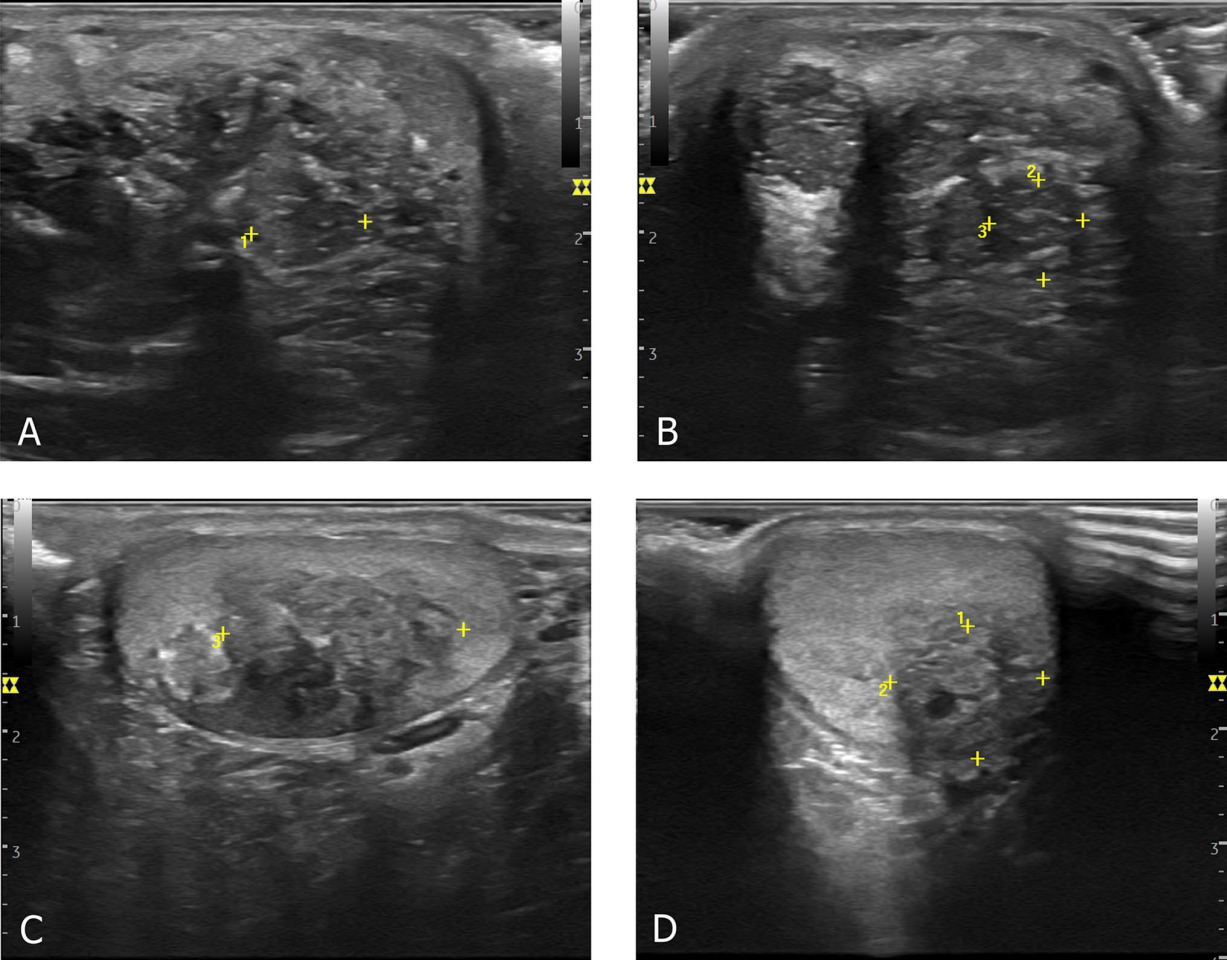
Ultrasound pictures of the testicular adrenal rest tumors. Upper panels: longitudinal (**A**) and transversal (**B**) scans of the right testis. Lower panels: longitudinal (**C**) and transversal (**D**) scans of the left testis

We added to our database the case of a patient not previously described in the literature who resulted in a carrier of compound heterozygous c.1891G > A, p. (Val631Ile) and c.516G > A variant. He had classical salt-wasting CAH, TARTs (Fig. 1), infertility, abnormal sperm parameters (oligoasthenoteratozoospermia, OAT), and extremely elevated ACTH serum levels. He achieved spontaneous paternity after adding dexamethasone 0.25 mg/day to his daily cortisol replacement therapy. This therapeutic arrangement led to the normalization of ACTH serum levels.

## Results

**Fig. 2:**
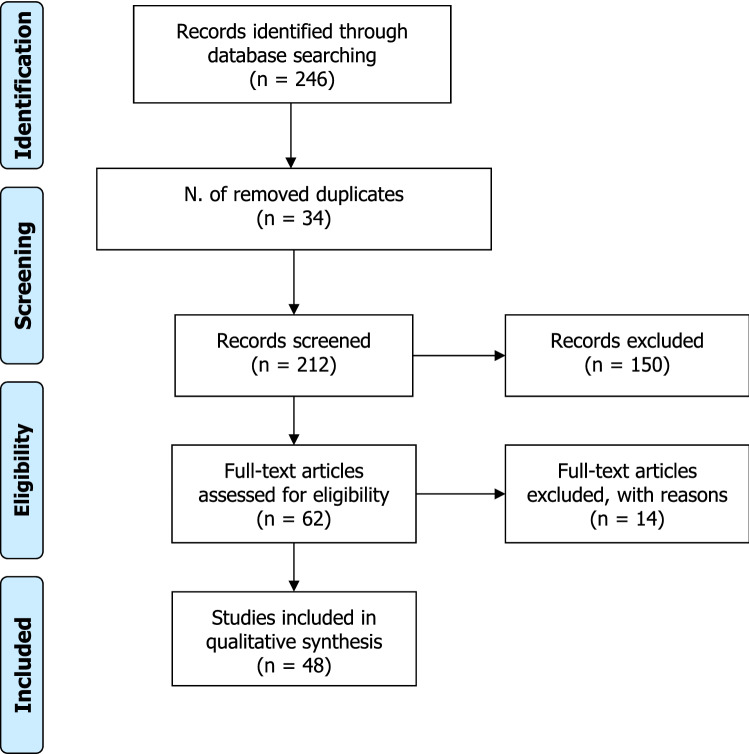
Flowchart of the included studies

Using the above-mentioned search terms, we found 246 articles. After the exclusion of 34 duplicated records, 212 articles were screened. Thirty-one articles were excluded because the English full-text was not available. Of the remaining articles, 119 were excluded after having read their title and abstract, since they did not satisfy the inclusion criteria. In particular, 10 studies were excluded because performed in vitro and/or on animals, and 24 were excluded because they were reviews. The remaining 62 full-texts were carefully read. Finally, 48 articles matched the inclusion criteria [14, 22, 24–69]. These studies included 119 patients (46 males and 73 females) with PORD (Fig. 2). They had homozygous, combined, or heterozygous *POR* gene variants in 58.0% (69/119), 32.8% (39/119), and 9.2% (11/119), respectively. These patients are worldwide distributed. The main features of the included studies are summarized in Table 1.

**Table 1:** Summary of the studies included in this systematic review

Authors	Study design	Sample size (M/F)	Age of patients (years)	POR genotype	CAH	DSD	TARTs	Fertility
Arlt et al. [24]	Case series and in-vitro study	1/2	2–14	Compound Het. (the authors did not indicate patient-specific mutations): c.531 T > G/p.Tyr178Asp c.849G > C/p.Ala284Pro c.1360G > A/p.Arg454His c.1696G > A/Cys566Tyr	Y (XX) Y (XY) Y (XX)	Y N Y	NR N NR	Unsearched NR NR
Fluck et al*.* [25]	Case series and in-vitro study	2/2 XX XY XY XX	5 4.5 1.9 23	Het: c.1370G > A(p.R457H)/731 + 1G > A Het: c.1475 T > A(p.V492E) Homo: c.859G > C (p.A287P) Het: c.1706G > A(p.C569Y)/c.1822G > T(V608F)	Y Y Y Y	Y Y Y N	NR N N NR	Unsearched (all)
Adachi et al*.* [26]	Case series	1/1 XY XX	14 11	Het: 1329insC/R457H Het:1698insC/R454H	Y Y	N Y	N NR	Unsearched (all)
Wudy et al. [27]	Case report	1/0	18	Homo: exon 8, GCT > CCT /p.Ala284Pro	Y	N	N	Unsearched
Shackleton et al*.* [28]	Case series	1/1 XX XY	At birth At birth	Het: p.Y178D/p.C566Y Het: p.Y178D/p.C566Y	Y Y	Y N	NR N	Unsearched (all)
Huang et al*.* [22]	Cohort study (18/19 excluded for lack of data) and in-vitro	0/1 XX (patient 16)	NR	Het: c.859G > C (p.A287P)/-	Y	Y	NR	Unsearched
Fukami et al. [29]	Case series	4/6 XY XY XX XY XY XX XX XX XX XX	26 29 10.9 17.9 17.5 2 6.7 9.5 13.2 15	Het: c.1370G > A(p.R457H)/c.1835-1858del(p.L612_W620delinsR) Het: c.1370G > A(p.R457H)/c.1835-1858del(p.L612_W620delinsR) Het: c.1329_1330insC(I444fsX449)/c.1733A > G(p.Y578C) Het: c.1329_1330insC(I444fsX449)/c.1733A > G(p.Y578C) Het: c.15A > G(p.G5G)/c.1370G > A(p.R457H) Homo: c.1370G > A(p.R457H)/ c.1370G > A(p.R457H) Het: c.1370G > A(p.R457H)/- Homo: c.1370G > A(p.R457H)/c.1370G > A(p.R457H) Het: c.1329_1330insC(p.I444fsX449)/c.1370G > A(p.R457H) Homo: c.1370G > A(p.R457H)/c.1370G > A(p.R457H)	Y Y Y Y Y Y Y Y Y Y	Y Y Y N N Y Y Y Y Y	N N NR N N NR NR NR NR NR	Unsearched (all)
Fukami et al. [30]	Case series	1/2 XY XX XX	10 5 1	Homozygous: R457H/R457H Homozygous: R457H/R457H Homozygous: R457H/R457H	Y Y Y	N Y Y	N NR NR	Unsearched (all)
Homma et al. [31]	Case–control	5/2 XY XY XY XY XY XX XX	22 23 15 16 0.3 0.7 12	Homo: R457H/R457H Homo: R457H/R457H Het: R457H/Q201X Het: R457H/Q201X Het: R457H/A462_S463insIA Het: R457H/E580Q Het: R457H/Q201X	Y all	N N N N Y Y N	N N N N N NR NR	
Williamson et al*.* [32]	Case report	0/1	NR	Het: p.A278P/p.H604P	Y	Y	NR	Unsearched
Hershkovitz et al*.* [33]	Case series	4/0 XY XY XY XY	21 14.6 16.5 At birth	Homo: c.1697G > A(p.G539R) Homo: c.1697G > A(p.G539R) Homo: c.1697G > A(p.G539R) Homo: c.1697G > A(p.G539R)	Y Y Y Y	Y Y Y Y	N N N N	Unsearched (all)
Nakamura et al*.* [34]	Case series (2/3 duplicated)	0/1	8	Het: p.T228I	Y	Y	NR	Unsearched
Ko et al. [35]	Case report	0/1	0.58	Het: c.1329_1330insC(I444fsX449)/ c.1370G > A (R457H)	Y	Y	NR	Unsearched
Sahakitrungruang et al. [36]	Case series	1/3 XY XX XX XX	3 24 21 3.5	Het: delGGA651-653(delE217)/859G > C(A287P) Het: 555T > A(N185K)/1730T > G(L577R) Het: 1615G > A(G539R)/1363delC Het: 1615G > A(G539R)/697-698insGAAC	Y Y Y Y	Y N Y Y	N NR NR NR	Unsearched Infertile Unsearched Unsearched
Fukami et al. [37]	Cohort study (23/35 duplicated, 11/12 lack of data)	1/0	13.1	Het: c.1370G > A(p.R457H)/(-)(DeltaExons 2–13)	Y	Y	N	Unsearched
Iijima et al. [38]	Case report	0/1	2.5	Het: 348delV/R457H	Y	N	NR	Unsearched
Idkowiak et al. [39]	Case report and in-vitro study	1/0	9	Het:c.32062delG(p.E601fsX12)/ c.32171A > G(p.Y607C)	Y	Y	N	Unsearched
Tomalik-Scharte et al. [40]	Case report	0/1	48	Homo: c.852G > C	Y	Y	NR	Unsearched
But et al*.* [41]	Case report	0/1	10	Homo:c.1370G > A/p.R457H	Y	Y	NR	Unsearched
Idkowiak et al*.* [42]	Case series	2/4 XX XX XX XX XY XY	12 23 19 16 16 13.5	Homo: A287P/A287P Homo: A287P/A287P Het: T142A/Y376LfsZ74 Het: A287P/R223X Het: R457H/Y576X Het: A287P/IVS7-dupT	Y Y Y Y Y Y	Y Y N Y N N	NR NR NR N N	Unsearched (all)
Fluck et al. [43]	Case series	0/2 XX XX	At birth At birth	Homo: c.1196_1204delCCTCGGAGC (p.Pro399_Glu401del)	Y Y	Y Y	NA (all)	Unsearched (all)
Herkert et al. [44]	Case report	0/1	19	Het:c.2640A > G, p.T142A c.30843dupC, p.Y376LfsX74	Y	N	NR	Unsearched
Krone et al*.* [45]	Cohort study (6/30 duplicated, 2/24 no *POR* mutation)	9/13 XX XX XY XY XY XX XX XX XX XY XY XX XY XX XX XY XX XX XY XY XX XX	At birth At birth At birth At birth At birth 12 At birth At birth At birth At birth At birth At birth At birth 24 At birth At birth At birth 18 At birth 12 16 31	Het: p.A287/- Het: A287P/ IVS6-2A > T Het: A287P/ IVS6-2A > T Het: A287P/V472AfsX102 Het: Q455RfsX544/IVS7 + 2dupT Homo: A287P/ A287P Het: A287P/DelexU1-1 Het: A287P/IVS8 + 1G > A Homo: A287P/ A287P Homo: A287P/ A287P Het: A287P/G188_V191dup Homo: A287P/ A287P Het: A287P/I444HfsX6 Homo: A287P/ A287P Het: Y87X/ - Het: R457H/Y576X Homo: R498P/R498P Het: Y376LfsX74/T142A Het: A287P/R616X Het: A287P/IVS7 + 2dupT Het: A287P/R223X Het: A287P/Dup ex 2–5	Y (all)	Y N Y Y Y Y N Y Y N Y Y Y Y Y Y Y N N N Y Y		Unsearched (all)
Boia et al. [46]	Case report	1/0	3	Homo: c.859G > C	Y	Y	N	Unsearched
Guaragna-Filho et al. [47]	Case report	0/1	9	Het: p.Arg223*/p.Met408Lys	Y	Y	NR	Unsearched
Sànchez-Garvìn et al*.* [48]	Case report	0/1	7	Het:c.1615G > A (p.Gly539Arg) p.Gly80Arg	Y	Y	NR	Unsearched
Nakanishi et al. [49]	Case report	0/1	At birth	Homo:p.R457H/ p.R457H	Y	Y	NR	Unsearched
Oldani et al. [50]	Case report	0/1	IUD	Het: c.859G > C(p.Ala287Pro)/c.732A > T	Y	Y	NR	NA
Koika et al. [51]	Case report	1/0	36	Het.:c.1591_1593delGTC(p.del531Val)/G858C,A259G rs1057868(C/TA503V) rs1057870 (G/AS572S)	Y	Y	NR	Unsearched
Parween et al. [52]	Case report and in-vitro study	0/1	At birth	Het:p.L374H/c.5 + 4A > G	Y	Y	NR	Unsearched
Bonamichi et al. [53]	Case series	0/1	15	Homo:p.A287P/p.A287P	Y	Y	NR	Unsearched
Tzetis et al. [54]	Case series (2 samples excluded for lack of data)	0/1	Fetus	Homo:c.859G > C	Y	Y	NR	Unsearched
Woo et al. [55]	Case report	1/0	7	Het: p.Arg457His/p.Gln555Profs19	Y	N	N	Unsearched
Bai et al*.* [56]	Case report	0/1	27	Homo: c.1370G > A(p.R457H)/c.1370G > A(p.R457H)	Y	Y	NR	Unsearched
Song et al*.* [57]	Case report	0/1	28	Het: c.976 T > G(p.Y326D)	Y	Y	NR	Y (ART)
Khadilkar et al. [58]	Case report	0/1	17	Het: c.430G > A(p.G144S)/c.1265G > A(p.W422X)	Y	Y	NR	Unsearched
Oh et al. [59]	Case report	0/1	21	Het:c.1370G > A (p.Arg457His); c.529G > C (p.Gly177Arg)	Y (mild phenotype)	Y (hypoplastic uterus)	NR	Unsearched
Fan et al. [60]	Case series	5/3 XX XY XY XY XY XX XY XX	4.2 2.2 1 0.5 3.5 17.8 9.8 12.5	Het: c.1370G > A(p.R457H)/c.744C > G(p.Y248X) Het:c.1370G > A(p.R457H)/c.744C > G(p.Y248X) Het:c.1370G > A(p.R457H)/c.1660C > T(p.R554X) Het:c.1370G > T(p.R457L)/c.1820A > G(p.Y607C) Het:c.1370G > A(p.R457H)/c.629A > G(p.D210G) Het:c.1370G > A(p.R457H)/c.517-19_517-10delGGCCCCTGTGinsC Het:c.1370G > A(p.R457H)/c.517-19_517-10delGGCCCCTGTGinsC Homo: c.1370G > A(p.R457H)/c.1370G > A(p.R457H)	Y Y Y Y Y Y Y Y	Y Y Y Y Y Y Y Y	NR N N N N NR N NR	Unsearched (all)
Lee et al*.* [61]	Case series (1/4 duplicated)	2/1 XX XY XY	5.3 3.3 2.6	Homo: c.1370G > A(p.R457H)/c.1370G > A(p.R457H) Homo: c.1370G > A(p.R457H)/c.1370G > A(p.R457H) Het:c.1329_1330insC(p.I444Hfs*6)/c.1370G > A(p.R457H)	Y Y Y	Y Y Y	NR N N	Unsearched (all)
Papadakis et al. [62]	Case series	0/5 XX XX XX XX XX	30 36 33 38 19	Het: c.1249-IG > C(p.?)/c.1324C > T(p.Pro442Ser) Het: c.1825C > T(p.Gln609*)/c.1859G > C(p.Trp620Ser) Het: c.1825C > T(p.Gln609*)/c.1859G > C(p.Trp620Ser) Het: c.1648C > T(p.Arg550Trp)/- Homo: c.859G > C(p.Ala287Pro)/c.859G > C(p.Ala287Pro)	Y Y Y Y Y	N N Y N N	NR (all)	Y (ART) Y (ART) Unsearched Unsearched Unsearched
Parween et al. [14]	Case report	0/1 XX	11	Het:c.73_74delCT(p.L25Ffs*93)/c.1648C > T(p.R550W)	Y	Y	NR	Unsearched
Zhang et al*.* [63]	Case report	0/1 XX	35	Het:c.1195_1203delCCTCGGAGC(p.399_401delPSE)/ IVS14-1G > G/C	Y	Y	NR	Y (ART)
Aljabri et al. [64]	Case report	1/0 XY	2.5	Compound: Homo: nonsense Het c.317A > G(p.His106Arg)	Y	N	N	Unsearched
Song et al*.* [65]	Case report	0/1 XX	10.5	Compound: Homo c.1370G > A(p.R457H) Het c.2978T > A(p.I993N)	Y	Y	NR	Unsearched
Unal et al. [66]	Case series	1/1 XX XY	8.5 1.25	Homo:c.929_937delTCTCGGACT(p.Ile310-Ser313delinsThr Homo:c.929_937delTCTCGGACT(p.Ile310-Ser313delinsThr	Y Y	Y N	NR N	Unsearched (all)
Wang et al*.* [67]	Case series	0/1 XX	31	Het: p.Arg223Ter/p.Tyr67Cys	Y	N	NR	Infertility
Rakover et al. [68]	Case series	0/1 XX	11.8	Homo: c.1615G > A(p.G539R)	Y	Y	NR	Unsearched
Pan et al*.* (69)	Case report	0/1 XX	29	Het: c.1370G > A(p.Arg457His)/c.1196_1204del(p.Pro399_Glu401del)	Y	N	NR	Y (ART)
Patient from the present study	Case report and literature systematic review	1/0	17	Het: c.1891G > A, p. (Val631Ile); c.516G > A	Y	N	Y	Y (spontaneous)

*ART* assisted reproductive technique, *CAH* congenital adrenal hyperplasia, *DSD* disorders of sex development, *F* female, *Het* heterozygous, *Homo* homozygous, *M* male, *N* no, *NR* not reported, *TARTs* testicular adrenal-rest tumors, *Y* yes, *IUD* intrauterine death

### Congenital adrenal hyperplasia

The results showed that all the 119 patients with PORD had CAH. All of them showed increased serum 17α-hydroxyprogesterone levels at baseline or after the ACTH-stimulation test. Hence, no difference in the prevalence of CAH among homozygous, combined, or heterozygous *POR* gene pathogenetic variant was found (Table 1).

### Disorder of sex development

DSD was described in 85 out of the 119 patients (71.4%) but there was a different genotype (homozygous or heterozygous) and gender-related distribution. DSD was found in 65.2% (45/69) of patients with compound heterozygous variants, 82.1% (32/39) of patients with homozygous variants, and 72.7% (8/11) of those with monoallelic heterozygous variants. Moreover, DSD occurred more frequently in females affecting 79.5% (58/73) of them and 58.7% (27/46) male patients (*p* < 0.05).

### Testicular adrenal rest tumors

Out of 119 patients retrieved in the literature, ultrasound evaluation was performed only in 15 male probands without evidence of TARTs. Considering the case reported here, the prevalence of TARTs in PORD is, therefore, 6.6% (1/15), taking into account only patients who have undergone testicular ultrasound screening.

### Fertility

Considering the higher prevalence of pediatric probands found in the literature, we found an important limitation in the assessment of fertility in patients with PORD. Of these, only 27 probands were over the age of 18 and, therefore, eligible for fertility research. No studies had focused on fertility in patients with PORD until 2017. To date, only five studies of female patients with PORD who have successfully delivered after ART-induced pregnancy have been published [57, 62, 63, 69].

## Discussion

By reviewing data from 48 articles including 119 patients from around the world, the present study aims to show the gender- and genotype-related prevalence of CAH, DSD, TARTs, and infertility in patients with heterozygous or homozygous *POR* gene variants. Genotype–phenotype correlation is sometimes a complex and attempted association and is still a matter of research, considering the most recent studies that have focused on mutations of the *CYP21A2* gene [70, 71]. This review allows a better understanding of the PORD phenotype. Our results show that CAH can be caused by both homozygous and heterozygous *POR* gene variants. Among 119 patients DSD was found in 85 (71.4%), respectively, in 65.2% (45/69) of patients with compound heterozygous variants, 82.1% (32/39) of patients with homozygous variants (82.1%), and 72.7% (8/11) of those with monoallelic heterozygous variants. Furthermore, DSD had a higher frequency in females affecting 79.5% (58/73) and 58.7% (27/46) male patients (*p* < 0.05), indicating that DSD affects both homozygous and heterozygous carrier patients, although with a higher prevalence in the former.

Specifically analyzing the individual variants, the most frequent are c.859G > C (p.A287P), typically with a higher prevalence of DSD when the heterozygous mutation is found in association with another monoallelic variant (DSD present in 81% of probands with compound heterozygous variants); c.1370G > A(p.R457H), that shows a higher prevalence of DSD when the homozygous variants are found (79%); c.1697G > A(p.G539R), with no difference between homozygous or heterozygous pathogenic variants (DSD present in 100% of probands) (Table 2). All the other detected variants, including the one found in our proband, were found in 1–2 patients (for each variant), so statistical analysis could not be applied (Table 3). According to a recent publication on *POR* polymorphisms, variants of this gene can influence CAH phenotypical expression acting as a genetic modifier of CYP21A2 defects [72]. Although the literature has so far focused on different allelic mutations on a single gene, we should now consider the coexistence of *POR* variants and polymorphisms in CAH patients carrying *CYP21A2, CYP11B1, CYP17A1, HSD3B2, StAR*, or *CYP11A1* variants.

**Table 2:** Prevalence of disorders of sex development (DSD) in homozygous and heterozygous pathogenetic variants

Mutation	Homozygous mutations	DSD in homozygous (%)	Heterozygous mutations	DSD in heterozygous (%)	Compound heterozygous mutations	DSD in compound heterozygous mutations (%)
c.859G > C (p.A287P)	12	75	3	66	16	81
c.1370G > A(p.R457H)	14	79	4	25	23	74
c.1697G > A(p.G539R)	5	100			2	100

**Table 3:** Single pathogenetic variant associations of P_450_ oxidoreductase-associated congenital adrenal hyperplasia

Mutation	Homozygous mutations	Heterozygous mutations	Composed heterozygous mutations
c.1706G > A(p.C569Y)/c.1822G > T(V608F)			1
GCT > CCT (p.Ala284Pro)	1		
p.Y178D/p.C566Y			2
c.1733A > G(p.Y578C)			2
p.T228I		1	
555T > A(N185K)/1730T > G(L577R)			1
c.32062delG(p.E601fsX12)/ c.32171A > G(p.Y607C)			1
c.852G > C	1		
T142A/Y376LfsZ74			1
p.Pro399_Glu401del	2		
c.2640A > G, p.T142A			2
Q455RfsX544/IVS7 + 2dupT			1
Y87X		1	
R498P	1		
p.Arg223Ter			2
c.1615G > A (p.Gly539Arg)			1
p.del531Val/G858C,A259G rs1057868(C/TA503V) rs1057870 (G/AS572S)			1
p.L374H/c.5 + 4A > G			1
c.976T > G(p.Y326D)		1	
c.430G > A(p.G144S)/c.1265G > A(p.W422X)			1
c.1249-IG > C(p.?)/c.1324C > T(p.Pro442Ser)			1
c.1825C > T(p.Gln609*)/c.1859G > C(p.Trp620Ser)			2
c.1648C > T(p.Arg550Trp)		1	1
c.1195_1203delCCTCGGAGC(p.399_401delPSE)/IVS14-1G > G/C			1
c.317A > G(p.His106Arg)		1	
c.929_937delTCTCGGACT(p.Ile310-Ser313delinsThr	2		
p. (Val631Ile); c.516G > A			1

This is the first article reporting the presence of TARTs in a patient with CAH due to PORD. So far, TARTs have been described in patients with 21α-OHD and 11ß-hydroxylase deficiency [73, 74]. It is thought that poor hormonal control, leading to high blood levels of ACTH, is an important factor in the pathogenesis of TARTs by inducing hypertrophy and hyperplasia of these adrenal-like cells within the testis [75, 76]. Accordingly, the case herein reported showed that TARTs tend to grow when ACTH levels are elevated. However, TARTs are also found in properly treated patients, whereas some poorly controlled CAH patients never develop TARTs despite they are chronically exposed to elevated ACTH levels [77, 78]. The most plausible explanation for this observation is that in the embryological period aberrant adrenal cells do not nestle in the testes in all males, so the presence of these aberrant adrenal cells within the testis is a prerequisite for the development of TARTs [73]. This ectopic migration does not seem to be related to a specific genotype as so far described. However, the presence of TARTs in the patients described in this article allows us to speculate that also *POR* gene variants, can cause TART development. Articles on PORD patients do not report the presence of TARTs in any of the patients described.

The present study shows, for the first time, spontaneous fatherhood in a patient with CAH and TARTs due to variants of the *POR* gene, although paternity must to be genetically proven once the child is born. Female patients, on the other side, may have infertility due to increased androgen secretion or impaired sex steroid production [79], but few cases of successful births by ART-induced pregnancy have been reported [57, 62, 63, 69]. This observation suggests that women with PORD must undergo ART programs to achieve pregnancy.

In conclusion, the unknown incidence of *POR* gene variants and the poorness of fertility-investigating reports enlighten that it is still unclear whether PORD is associated with human infertility since most of the cases reported so far did not focus on patients’ fertility. Further studies exploring the relationship between POR genotype and fertility are needed. Evidence from the male proband herein reported suggests that spontaneous fatherhood can occur in male patients with PORD but proper management of CAH is needed to reach spontaneous fertility without the need to undergo ART.
